# Decision-making about antidepressant medication use in pregnancy: a comparison between women making the decision in the preconception period versus in pregnancy

**DOI:** 10.1186/s12888-020-2478-8

**Published:** 2020-02-07

**Authors:** Lucy C. Barker, Cindy-Lee Dennis, Neesha Hussain-Shamsy, Donna E. Stewart, Sophie Grigoriadis, Kelly Metcalfe, Tim F. Oberlander, Carrie Schram, Valerie H. Taylor, Simone N. Vigod

**Affiliations:** 1 Department of Psychiatry, University of Toronto, Toronto, Ontario M5T 1R8 Canada; 2 Institute for Health Policy, Management and Evaluation, University of Toronto, Toronto, Ontario M5T 3M6 Canada; 3 Department of Psychiatry, Women’s College Hospital and Research Institute, Toronto, Ontario, 76 Grenville street Rm. 7234, Toronto, ON M5S 1B2 Canada; 4 Lawrence S. Bloomberg Faculty of Nursing, University of Toronto, Toronto, Ontario M5T 1P8 Canada; 5 Li Ka Shing Knowledge Institute, St. Michael’s Hospital, Toronto, Ontario M5B 1T8 Canada; 6 Department of Psychiatry, University Health Network, Toronto, Ontario M5G 2C4 Canada; 7 Department of Psychiatry, Sunnybrook Health Sciences Centre, Toronto, Ontario M4N 3M5 Canada; 8 Department of Pediatrics, University of British Columbia, Vancouver, British Columbia V6H 3V4 Canada; 9 Department of Family and Community Medicine, University of Toronto, Toronto, Ontario M5G 1V7 Canada; 10 Hannam Fertility Clinic, Toronto, Ontario M4W 3R2 Canada; 11 Present address: Department of Psychiatry, University of Calgary, Calgary, Alberta Canada

**Keywords:** Antidepressant medications, Decision making, Perinatal depression, Preconception, Pregnancy

## Abstract

**Background:**

Decisions about antidepressant use in pregnancy are complex. Little is known about how pregnancy-planning and already pregnant women making these decisions differ.

**Methods:**

In 95 Canadian women having difficulty deciding whether to take antidepressants in pregnancy, we compared sociodemographic factors, clinical characteristics, and treatment intent between women planning pregnancy (preconception women) and currently-pregnant women.

**Results:**

About 90% of preconception women (*n* = 55) were married or cohabitating and university-educated, and over 60% had an annual income of > 80,000 CAD/year; this was not different from currently-pregnant women (*n* = 40). Almost all women had previously used antidepressants, but preconception women were more likely to report current use (85.5% vs. 45.0%). They were more likely to have high decisional conflict (83.6% vs. 60.0%) and less likely to be under the care of a psychiatrist (29.1% vs. 52.5%). Preconception women were more likely than pregnant women to report the intent to use antidepressants (60% vs. 32.5%, odds ratio 3.11, 95% confidence interval 1.33–7.32); this was partially explained by between-group differences in current antidepressant use.

**Conclusions:**

Preconception women were more likely than pregnant women to intend to use antidepressants in pregnancy, in part because more of them were already using this treatment. Strategies to enhance support for decision-making about antidepressant medication use in pregnancy may need to be tailored differently for pregnancy-planning and already pregnant women.

## Background

Depression occurs in up to 1 in 5 women, disproportionately affecting them during their reproductive years [1, 2]. In pregnancy, untreated or under-treated depression can lead to adverse maternal and child outcomes including premature delivery, decreased breastfeeding initiation, and cognitive, emotional, and behavioural problems in children [3, 4]. It can also lead to postpartum depression, a condition with serious consequences for women, children, and families [5]. While many women experience remission of depression with psychological treatments, some require medication and must decide whether to start or continue antidepressant medications in pregnancy. While antidepressants are not major teratogens, they are linked to small increased risks for cardiac malformations, spontaneous abortion, respiratory distress, tremors, and neurodevelopmental problems, so careful consideration of the risks and benefits of their use in pregnancy is required [6–10]. Decisions about whether to take antidepressant medications in pregnancy can be complex, especially because of this residual uncertainty pertaining to the benefits and risks.

Previous studies on pregnancy-related antidepressant decisions have been restricted mainly to pregnant or recently pregnant women [11–18]. However, the preconception period is also important to consider. Two studies of large health administrative databases found that over 6% of women in North America are prescribed antidepressant medication in the year prior to pregnancy, with the most recent study finding that 6.3% of pregnant women filled an antidepressant prescription in the 90 days before conception alone [19, 20]. Making a decision about the treatment of depression before conception may better optimize maternal mental health and pregnancy outcomes compared to waiting until pregnancy onset [6, 21, 22]. This could prevent the abrupt discontinuation of antidepressants when women learn that they are pregnant (which is associated with a high risk of depression relapse), and/or ensure that women who need antidepressants for treatment of depression have the opportunity to remit prior to pregnancy [23, 24]. Despite their high rates of depression and antidepressant use, there has been little focus on preconception women, and their plans regarding antidepressant use in pregnancy.

In this study of women having difficulty deciding whether or not to use antidepressant medication in anticipation of, or during, a pregnancy, we aimed to understand and compare the demographic, clinical, and decisional characteristics of preconception and pregnant women to help inform us about whether supports and services might need to be tailored differently depending on a woman’s pregnancy status.

## Methods

### Study design

This study used baseline cross-sectional (i.e. prior to randomization and prior to intervention) data from a clinical trial of an online patient decision aid (PDA) for preconception and pregnant women having difficulty deciding whether to either start or continue antidepressant medication in anticipation of, or during, a pregnancy (ClinicalTrials.gov registration number: NCT02308592) [25]. In this study, we characterized preconception participants on sociodemographic, clinical, and health service utilization factors. We then determined the proportion of preconception participants who indicated that they intended to start or continue antidepressant medications in preparation for pregnancy, and compared this to the proportion of already-pregnant patients with the same intent. The parent trial was approved by the Research Ethics Board at Women’s College Hospital.

### Participants

Participants were recruited for the parent trial between January 2015 and February 2017 through physician referrals from the Reproductive Life Stages program at Women’s College Hospital (a specialized perinatal mental health program) and through online recruitment from across Canada (social media and the Women’s College Hospital website). Under the supervision of the principal investigator (SV), trained research personnel confirmed eligibility and reviewed consent processes in-person or by phone, obtained written informed consent, and collected data online or by phone. Women were eligible for the trial if they were aged ≥18 years, preconception (planning a pregnancy within the upcoming 1 year) or pregnant (< 30 weeks gestation to allow for an implemented decision within the pregnancy), diagnosed with major depressive disorder by a clinical provider (confirmed using the Mini Neuropsychiatric Interview administered by research personnel over the phone [26]), and actively deciding whether to start or continue selective serotonin reuptake inhibitor (SSRI) or serotonin-norepinephrine reuptake inhibitor (SNRI) medication. Because the aim of the intervention for the parent trial was to reduce decisional conflict and improve decision effectiveness, only women who were experiencing moderate to high decisional conflict were eligible for the trial (Decisional Conflict Scale ≥25, DCS, range 1–100) [27–29].

### Descriptive characteristics

We collected data on age, marital status, educational attainment, household income, country of birth, parity (previous births), medical comorbidities, smoking, lifetime history of antidepressant medication use (including response and side effects), and prior mental health service use. We assessed participants’ current use of mental health services and treatments, including the type of physician involved in their mental health care (psychiatrist, family physician, both, or none), engagement in individual and/or group psychotherapy, and antidepressant use. Active depressive symptomatology was assessed using the Edinburgh Postnatal Depression Scale (EPDS), a self-report screening tool validated in pregnancy (range 0–30, scores ≥13 indicate active depressive symptoms) [30] and the State-Trait Anxiety Inventory (STAI), which consists of two self-report screening scales for “state” and “trait” anxiety and has been validated in perinatal women (range 20–80 for each scale, scores ≥40 on the state-STAI suggest clinically significant anxiety) [31].

While all women in this study had at least moderate decisional conflict (DCS ≥ 25), scores ≥37.5 suggest a much higher likelihood of delayed decision-making and feeling unsure about decision implementation, so DCS score was also a covariate [28, 29]. To assess knowledge of treatment options for depression in pregnancy (benefits and risks of psychotherapy, medication, and no treatment), a questionnaire was modified from a knowledge tool used previously by the investigators [25, 32].

### Primary outcome

All women were asked which way they were leaning in terms of their intent to use an antidepressant in pregnancy, i.e. to (a) use antidepressant medication in pregnancy, or (b) not use antidepressant medication in pregnancy.

### Statistical analysis

Baseline descriptive characteristics were compiled for participants, and were compared between preconception and pregnant women using chi-square tests (categorical variables) and independent samples t-tests (continuous variables). We then characterized women who were intending to and not intending to use antidepressants in pregnancy in relation to each descriptive variable and compared groups using t-tests and chi-square tests. For the main analysis, we compared the intent to use antidepressant medications in pregnancy between preconception and pregnant participants. We compared the likelihood of intent to use antidepressants between preconception and pregnant women (referent group) using logistic regression, generating an odds ratio (OR) and 95% confidence interval (CI). The model was then adjusted for the variables that were significantly associated with intent to use antidepressants on univariate analysis (*p* < 0.05). Models were assessed for collinearity (variance inflation factor > 2.5). We used Nagelkerke R-squared to estimate the variance in intent to use antidepressant medications explained by the models.

In additional analyses, we performed analyses stratified on: (1) age (under < 35 years; ≥35 years), (2) parity (no previous births; previous births), and (3) depressive symptom status (EPDS < 13; EPDS ≥13) as prior literature suggests that these factors may interact with preconception/pregnancy status and treatment decision-making [30, 33–35]. We also planned to stratify on type of decision (i.e. whether to start or continue) an antidepressant, as the decision might be expected to differ between these levels of that variable. Statistical analyses were conducted using SPSS Versions 24.0 and 26.0.

## Results

There were 96 women enrolled in the parent trial, one of whom was excluded from the current study as she did not report on her intent with respect to antidepressant use. Of the 95 included participants, 55 (57.9%) were preconception, and 40 (42.1%) were pregnant at enrollment (Table 1). Mean age was 33.6 years (standard deviation, SD 4.22). Most women were married or living with a partner (*n* = 87, 91.6%), university educated (*n* = 91, 95.8%), in the highest income bracket (*n* = 58, 61.1%), and Canadian-born (*n* = 82, 86.3%).

**Table 1:** Baseline characteristics of preconception women and pregnant women with high decisional conflict. Categorical variables presented as n (%) and compared using chi-squared (*x*^*2*^) tests, and continuous variables presented as mean ± standard deviation (SD) and compared using independent t-tests

Variable	Preconception (*n* = 55)	Pregnant (*n* = 40)	Test statistic; *P*-value
Recruitment Site (Online), n (%)	30 (54.5)	15 (37.5)	*x*^*2*^ = 2.70; *p* = 0.10
Demographics and Health History			
Age in years, Mean ± SD	33.3 ± 4.29	33.9 ± 4.15	t = 0.68; *p* = 0.50
Married or cohabitating with partner, n (%)	51 (92.7)	36 (90.0)	*x*^*2*^ = 0.22; *p* = 0.64
Completed a university degree, n (%)	52 (94.5)	39 (97.5)	*x*^*2*^ = 0.50; *p* = 0.48
Annual household income (CAD/year), n (%)			*x*^*2*^ = 4.22; *p* = 0.38
< 40,000	2 (3.6)	5 (12.5)	
40,000-80,000	16 (29.1)	13 (32.5)	
> 80,000	36 (65.5)	22 (55.0)	
Preferred not to answer	1 (1.1)	0 (0)	
Canadian-born, n (%)	49 (89.1)	33 (82.5)	*x*^*2*^ = 2.14; *p* = 0.34
Nulliparous, n (%)	37 (67.3)	23 (57.5)	*x*^*2*^ = 0.95; *p* = 0.33
Current smoker, n (%)	4 (7.3)	3 (7.5)	*x*^*2*^ = 0.002; *p* = 0.97
Other medical condition, n (%)	20 (37.0)	14 (35.0)	*x*^*2*^ = 0.04; *p* = 0.84
Psychiatric History			
Lifetime antidepressant use, n (%)	55 (100.0)	36 (90.0)	*x*^*2*^ = 4.37; *p* = 0.04
Prior response to antidepressants, n (%)^a^			*x*^*2*^ = 5.62; *p* = 0.06
No effect	1 (1.8)	5 (14.3)	
Moderate effect	15 (27.3)	10 (28.6)	
Beneficial effect	39 (70.9)	20 (57.1)	
Prior adverse effects from antidepressants, n (%)^a^	21 (38.2)	16 (45.7)	*x*^*2*^ = 0.50; *p* = 0.48
Lifetime psychiatric hospitalization, n (%)	3 (5.5)	6 (15.0)	*x*^*2*^ = 2.46; *p* = 0.12
Current mental health treatment^b^			
Antidepressant medication, n (%)	47 (85.5)	18 (45.0)	*x*^*2*^ = 17.5; *p* < 0.001
Individual therapy, n (%)	20 (36.4)	17 (42.5)	*x*^*2*^ = 0.37; *p* = 0.55
Group therapy, n (%)	2 (3.6)	1 (2.5)	*x*^*2*^ = 0.10; *p* = 0.76
Psychiatrist, n (%)	16 (29.1)	21 (52.5)	*x*^*2*^ = 5.34; *p* = 0.01
Family doctor, n (%)	30 (54.5)	17 (42.5)	*x*^*2*^ = 1.34; *p* = 0.25
Social worker, n (%)	3 (5.5)	4 (10.0)	*x*^*2*^ = 0.70; *p* = 0.40
Psychologist, n (%)	11 (20.0)	4 (10.0)	*x*^*2*^ = 1.74; *p* = 0.19
Scales			
Edinburgh Postnatal Depression Scale, Mean ± SD	11.5 ± 4.76	13.4 ± 5.42	t = 1.85; *p* = 0.07
High Edinburgh Postnatal Depression Scale (≥13), n(%)	22 (40.0)	26 (65.0)	*x*^*2*^ = 5.79; *p* = 0.02
State-Trait Anxiety Inventory (Trait), Mean ± SD	50.0 ± 10.8	48.9 ± 10.5	t = −0.47; p = 0.64
High State-Trait Anxiety Inventory (Trait) (≥40), n(%)	45 (81.8)	32 (80.0)	*x*^*2*^ = 0.05; *p* = 0.82
State-Trait Anxiety Inventory (State), Mean ± SD	42.3 ± 12.2	46.3 ± 14.0	t = 1.49; *p* = 0.14
High State-Trait Anxiety Inventory (State) (≥40), n(%)	26 (47.3)	22 (55.0)	*x*^*2*^ = 0.55; *p* = 0.46
Decisional Conflict Scale, Mean ± SD	49.3 ± 14.2	42.5 ± 50.0	t = −2.27; *p* = 0.03
High Decisional Conflict Scale (≥37.5), n(%)	46 (83.6)	24 (60.0)	*x*^*2*^ = 6.67; *p* = 0.01
Knowledge Score, Mean ± SD	12.0 ± 1.50	12.1 ± 1.90	t = 0.34; *p* = 0.74

^a^Percentage of those who had used antidepressive agents. Note that prior benefit/side effects is missing for one pregnant participant

^b^These categories are not mutually exclusive

### Characteristics of preconception and pregnant women

Preconception and pregnant women were similar in terms of their sociodemographic characteristics (Table 1). Preconception women were more likely than pregnant women to have previously used antidepressants (100.0% vs. 90.0%). Of women who had previously used antidepressants, 70.9% of preconception women and 57.1% of pregnant women reported prior significant benefit, while 1.8% of preconception women and 14.3% of pregnant women reported no benefit at all; this difference was not statistically significant. Preconception women were much more likely be taking an antidepressant medication at the time of trial enrollment (85.5% vs. 45.0%, see Additional file 1: Table S1 for specific agents) and less likely to have active depression symptoms (40.0% vs 65.0%). They were much less likely to have a psychiatrist as part of their mental health care provision (29.1% vs. 52.5%), and more likely to have very high decisional conflict (DCS ≥ 37.5; 83.6% vs. 60.0%).

### Intent to use antidepressants

About half the participants overall (48.4%) intended to use antidepressant medication in pregnancy at the time of enrollment. Other than pregnancy status itself, characteristics significantly associated with increased likelihood of the intent to use antidepressant medication in pregnancy were: (1) being married or cohabitating with a partner, (2) current antidepressant use, [3] mental health care from a family physician, (4) lower EPDS score, (4) lower anxiety (state-STAI < 40), and (5) lower decisional conflict (DCS score < 37.5) (Table 2).

**Table 2:** Characteristics of women who intend to use antidepressant medication in pregnancy (*n* = 46) and those who do not intend to use antidepressant medication in pregnancy (*n* = 49). Categorical variables presented as n (%) and compared using chi-squared (*x*^*2*^) tests, and continuous variables presented as mean ± standard deviation (SD) and compared using independent t-tests

Variable	Intends to use antidepressant in pregnancy (*n* = 46)	Intends to not use antidepressant in pregnancy (*n* = 49)	Test statistic; *P*-value
Demographics and Health History			
Age in years, Mean ± SD	33.6 ± 4.3	33.6 ± 4.2	t = 0.02; *p* = 0.99
Married or cohabitating with partner, n (%)	45 (97.8)	42 (85.7)	*x*^*2*^ = 4.51; *p* = 0.03
Completed a university degree, n (%)	43 (93.5)	48 (98.0)	*x*^*2*^ = 1.18; *p* = 0.28
Annual household income (CAD/year), n (%)			*x*^*2*^ = 6.19; *p* = 0.18
< 40,000	1 (2.2)	6 (12.3)	
40,000-80,000	12 (26.1)	17 (34.7)	
> 80,000	32 (69.6)	26 (53.1)	
Preferred not to answer	1 (2.2)	0 (0.0)	
Canadian-born, n (%)	40 (87.0)	42 (85.7)	*x*^*2*^ = 1.29; *p* = 0.52
Nulliparous, n (%)	29 (63.0)	31 (63.3)	*x*^*2*^ = 0.001; *p* = 0.98
Current smoker, n (%)	3 (6.5)	4 (8.2)	*x*^*2*^ = 0.09; *p* = 0.76
Other medical condition, n (%)	12 (26.7)	22 (44.9)	*x*^*2*^ = 3.38; *p* = 0.07
Psychiatric History			
Lifetime antidepressant use, n (%)	44 (95.7)	47 (97.9)	*x*^*2*^ = 0. 39; *p* = 0.53
Prior response to antidepressants, n (%)^a^			*x*^*2*^ = 4.46; *p* = 0.11
No effect	1 (2.2)	5 (10.9)	
Moderate effect	10 (21.7)	15 (32.6)	
Beneficial effect	33 (71.7)	26 (56.5)	
Prior adverse effects from antidepressants, n(%)^a^	18 (40.9)	19 (41.3)	*x*^*2*^ = 0.001; *p* = 0.97
Lifetime psychiatric hospitalization, n (%)	5 (10.9)	4 (8.2)	*x*^*2*^ = 0.20; *p* = 0.65
Current mental health treatment^b^			
Antidepressant medication, n (%)	42 (91.3)	23 (46.9)	*x*^*2*^ = 21.6; *p* < .001
Individual therapy, n (%)	15 (32.6)	22 (44.9)	*x*^*2*^ = 1.51; *p* = 0.22
Group therapy, n (%)	2 (4.3)	1 (2.0)	*x*^*2*^ = 0.41; *p* = 0.52
Psychiatrist, n (%)	16 (34.8)	21 (42.9)	*x*^*2*^ = 0.65; *p* = 0.42
Family doctor, n (%)	29 (63.0)	18 (36.7)	*x*^*2*^ = 10.2; *p* < 0.01
Social worker, n (%)	1 (2.2)	6 (12.2)	*x*^*2*^ = 3.38; *p* = 0.07
Psychologist, n (%)	6 (13.0)	9 (18.4)	*x*^*2*^ = 0.41; *p* = 0.52
Scales			
Edinburgh Postnatal Depression Scale, Mean ± SD	11.1 ± 5.6	13.4 ± 4.3	t = −2.28; *p* = 0.02
High Edinburgh Postnatal Depression Scale (≥13), n(%)	20 (43.5)	28 (57.1)	*x*^*2*^ = 1.77; *p* = 0.18
State-Trait Anxiety Inventory (Trait), Mean ± SD	48.4 ± 12.0	50.5 ± 9.2	t = −0.95; *p* = 0.34
High State-Trait Anxiety Inventory (Trait) (≥40), n(%)	35 (76.1)	42 (85.7)	*x*^*2*^ = 1.43; *p* = 0.23
State-Trait Anxiety Inventory (State), Mean ± SD	41.9 ± 13.2	45.9 ± 12.8	t = −1.47; *p* = 0.15
High State-Trait Anxiety Inventory (State) (≥40), n(%)	18 (39.1)	30 (61.2)	*x*^*2*^ = 4.63; *p* = 0.03
Decisional Conflict Scale, Mean ± SD	43.7 ± 15.6	49.0 ± 13.6	t = − 1.74; *p* = 0.08
High Decisional Conflict Scale (≥37.5), n (%)	29 (63.0)	41 (83.7)	*x*^*2*^ = 5.21; *p* = 0.02
Knowledge Score, Mean ± SD	64.8 ± 8.4	64.6 ± 9.8	t = 0.43; *p* = 0.67

^a^Percentage of those who had used antidepressive agents. Note that prior benefit/side effects is missing for one participant who did not intend to take antidepressants

^b^These categories are not mutually exclusive

Preconception women were more likely to endorse the intent to use antidepressants than pregnant women (60.0% vs. 32.5%, crude OR 3.11, 95% CI 1.33 to 7.32, Nagelkerke *R*^2^ = 9.6%) (Table 3). In the model adjusted for the other factors associated with intent to use antidepressants, the point estimate was similar however the association was no longer significant and a much larger proportion of the variance in intent to use antidepressants was explained (adjusted OR 2.79, 95% CI 0.81 to 9.62; Nagelkerke *R*^2^ = 44.6%).

**Table 3:** Multivariable model for the relation between pregnancy status and intent to use antidepressants. Crude and adjusted models using odds ratios (OR) and 95% confidence intervals (CI)

Main Exposure Variable	Odds Ratio (95% CI)
Crude model^a^	
Preconception (vs. pregnant)	3.11 (1.33–7.32)
Adjusted model^b^	
Preconception (vs. pregnant)	2.79 (0.81–9.62)
Married/co-habitating (vs. single, divorced, widowed)	6.10 (0.58–64.7)
Current antidepressant use (vs. no current use)	12.4 (2.72–56.4)
Family physician involved in mental health care (vs. not)	1.32 (0.46–3.80)
Edinburgh Postnatal Depression Scale Score (Mean)	0.94 (0.82–1.07)
State-Trait Anxiety Inventory (State) ≥ 40 (vs. < 40)	0.62 (0.18–2.17)
Decisional Conflict Scale Score ≥ 37.5 (vs. < 37.5)	0.16 (0.04–0.67)

^a^ Nagelkerke R-squared 9.6%

^b^ Hosmer-Lemeshow Chi-Square = 3.83 (df 8), *p* = 0.872, Nagelkerke R-squared 44.6%

### Additional analyses

**Fig. 1:**
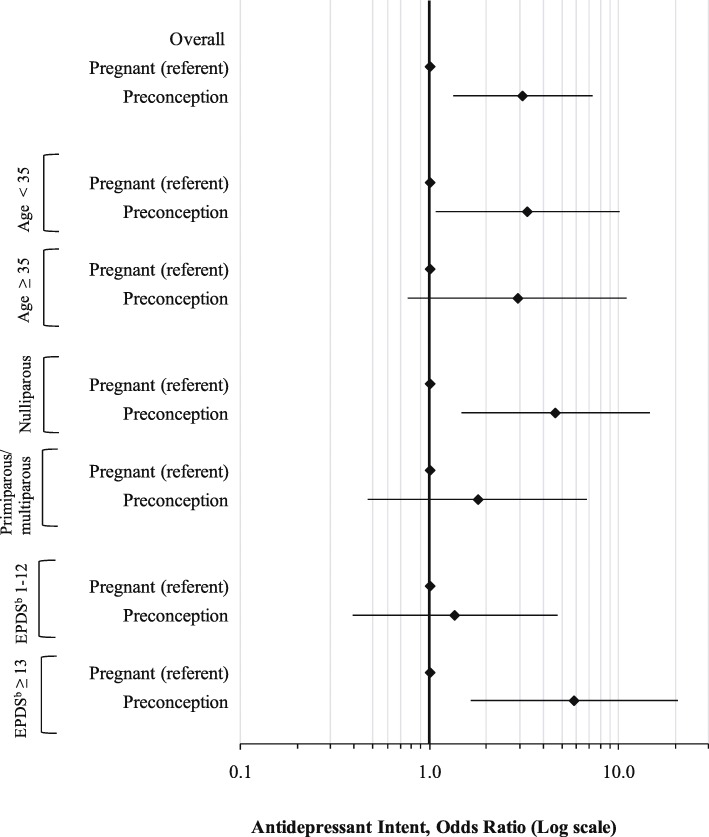
Unadjusted models^a^ showing the association between pregnancy status and antidepressant medication intent for women, stratified by select demographic and clinical characteristics, presented as odds ratios (OR) and 95% confidence intervals (CIs). ^a^Note that only unadjusted logistic regression models were generated for the stratified analyses due to limited sample size. ^b^EPDS = Edinburgh Postnatal Depression Scale

Percentages of preconception and pregnant women with intent to use antidepressants in pregnancy in the stratified analyses are presented in Additional file 1: Figure S1. In age-stratified analysis, the relation between pregnancy status and antidepressant intent was only significant for women aged under 35 years, with similar point estimates in both strata (Fig. 1). Among women with no prior births (nulliparous women), preconception women had an almost 5-fold higher odds of intent to use antidepressants compared to pregnant women (OR 4.65, 95% CI 1.48 to 14.6), whereas among women who had given birth previously the relation between pregnancy status and antidepressant intent was non-significant. Among women with active depressive symptoms (EPDS ≥13) preconception women had an almost 6-fold increased odds of intent to use antidepressants (OR 5.83, 95% CI 1.65 to 20.6); this association was non-significant in women without active depressive symptoms. We could not stratify based on decision type (i.e. starting vs. continuing the antidepressant) because almost all preconception women were taking antidepressants at baseline (*n* = 47, 85.5%) such that the stratified models would not converge.

## Discussion

In a sample of women having difficulty making a decision about antidepressant medication use in pregnancy, about 60% of preconception women reported that they intended to start or continue the medication, compared to only 32.5% of pregnant women faced with the same decision. Several other factors were associated with an increased likelihood of intent to use antidepressants in pregnancy: being married or cohabitating with a partner, current use of antidepressant medication, connection to a family physician, lower current depression score, lower anxiety, and lower decisional conflict. After accounting for these factors (especially preconception women’s greater use of antidepressants at the time of decision-making), the difference between preconception and pregnant women was not significant. Preconception women were, however, more conflicted about their decision and were less likely to be under the care of a psychiatrist than the pregnant women, which both suggest unmet need in regard to decision-making that is distinct for this group, compared to their pregnant counterparts. Since over 6% of preconception women face decisions about antidepressant use in pregnancy, access to specialized psychiatric care for each woman may not be a realistic endeavor [19, 20]. As such, finding efficient and scalable ways to better support antidepressant decision-making in general preconception care may be warranted.

The novel aspect of this study is its specific focus on pregnancy-related depression treatment plans in the preconception period. To our knowledge, two studies have reported on pregnant women’s intent regarding use of antidepressant medications. In a convenience sample of 509 predominantly well-educated, high-income, married pregnant (third trimester) women in the United States, Goodman [13] found that 33% would take antidepressant medication if recommended by their healthcare provider. This is virtually the same proportion as was found among the pregnant women in our study (32.5%), in a demographically very similar sample. This prior study also found that prior or current antidepressant medication use was the strongest determinant of preference for antidepressant use in pregnancy, as we did [13]. In the second study, Battle et al. (2013) conducted interviews with 61 pregnant women, half of whom were depressed, and found very low preference rates for antidepressant medication (8%) [12]. However, this study asked about preference, not intent to use, and only about first choice of treatment; combination options (e.g. medication with psychotherapy) were not included as choice options.

The greater proportion of preconception women than pregnant women who intended to use antidepressants in pregnancy was partially explained by the women’s experience with antidepressants. Over 85% of preconception participants were taking antidepressant medication (versus 45% of pregnant participants), and a greater proportion of them were well (EPDS < 13) at baseline, suggesting that they were currently benefitting from the medication; their higher intent to use antidepressants makes sense in this context. None of this is surprising given that it would make sense that women who were already taking antidepressants and wanting/planning to conceive would try to make their decision prior to conception, whereas more women making the decision while already pregnant may have had an unexpected pregnancy or a new-onset depressive episode and were having to consider antidepressants unexpectedly in the pregnancy. However, the results do suggest that the circumstances of women making decisions about antidepressant use in the preconception vs. in the pregnancy itself are probably quite distinct, and may require different healthcare approaches at the individual patient and system levels.

Preconception women in our study, who had higher decisional conflict than their pregnant counterparts, were less likely to be currently under the care of a psychiatrist. Although family physicians routinely manage mental health care, they often report perceived misinformation and concerns about liability with respect to antidepressant medications in pregnancy [36]. However, knowledge scores were similar in preconception and pregnant women, suggesting that the difference in decisional conflict between groups is not due to unequal access to evidence-based information. This raises the possibility that even when family physician advice is adequate and accurate, women do not feel adequately reassured by it without specialist input. Both family physicians and patients may feel more reassured if information is coming from a psychiatrist.

Primary care providers are responsible for much of both preconception and depression care, and primary care is therefore a natural setting for broad-reaching preconception depression care [37, 38]. It is important to address family physicians’ concerns and hesitancies with respect and support them in providing to depression care in pregnancy [36]. Support pathways to specialized advice for preconception women may need to be designed specifically to support primary care, as before women are pregnant they do not necessarily have access to obstetrical centres where highly specialized reproductive psychiatric services are often available to inform decision-making. This study uses baseline data from a trial of a patient decision aid tool, and this tool is an example of an intervention that could be beneficial to preconception women and their primary care providers [25]. One of the intents of the decision aid is to allow family physicians and patients to access expert and up to date information from perinatal psychiatrists to support decision-making.

In some (but not all) jurisdictions, pregnant women may be at an advantage in terms of access to psychiatric are as they are already connected to prenatal care specialists including obstetricians, midwives, and obstetrics-focused family physicians who may have specific expertise in this field and/or established pathways to access specialized resources. However, there are still major gaps in access to mental health care for pregnant women, and American research has found that as few as 12% of depressed pregnant women receive mental health care [39]. Even when a woman is referred to appropriate services, this does not necessarily mean that she receives adequate support. Collaborative and stepped care models are effective for depression treatment, and further research on integrating these approaches with preconception care may be a useful strategy [38]. Existing collaborative programs that support clinicians to provide preconception and pregnancy mental health, such as the Massachusetts Child Psychiatry Access Project for Moms in the United States which involves provider training, telephone consultation with perinatal psychiatrists, and care coordination, could be scaled up to provide perinatal mental health supports in more jurisdictions [40].

We found especially large effects of pregnancy status on antidepressant medication treatment intent among nulliparous women and among women with active depressive symptoms. Nulliparous preconception women were much more likely than their nulliparous pregnant counterparts to report the intent to use antidepressants in pregnancy. Women often have very high teratogenic risk perception during their first pregnancy, so this finding is not surprising [34]. It raises the question of whether preconception women intending to use antidepressants in pregnancy will change their minds when pregnant; preconception initiatives should consider this possibility and maintain a dynamic and flexible approach to treatment through the perinatal period. Also, preconception women with active depressive symptoms were much more likely to intend to use antidepressants in pregnancy than their actively depressed pregnant counterparts. While this could also be related to teratogenic risk perception, pregnant women with active depressive symptoms may be more readily referred to specialty perinatal mental health services than preconception women, and may therefore have more access to alternatives such as psychotherapy. This suggests potential gaps in care for women with active depressive symptoms who are planning pregnancy.

The main strength of this paper is our national sample of women actively making decisions about antidepressant use in pregnancy, including preconception women, and women from outside specialty healthcare settings, neither of whom have been well-characterized as it relates to this important clinical issue. We captured multiple sociodemographic, clinical, and health service use variables, including symptom scales that informed us about the needs and treatment preferences in this population. However, we only included women with at least moderate decisional conflict so this may not be representative of all women making pregnancy-related antidepressant decisions. Future research will also need to consider women who do not have difficulty deciding whether to take antidepressants in pregnancy, as they may be distinct from women with decisional conflict both in terms of the factors influencing their decision and their decisional support needs. We also did not distinguish between planned and unplanned pregnancies within the pregnant group. Although the decision of whether to continue maintenance antidepressants into pregnancy would ideally be made before conception, about half of pregnancies are unplanned, so decision-making issues may differ between these groups [24, 41]. Future research should consider the specific decisional support needs of women on antidepressants who may become pregnant unexpectedly. Another issue with respect to generalizability is that while the sample came from a geographically diverse area, the women in this study were mostly highly-educated, married, non-immigrant women who were motivated to participate in the clinical trial; our results may not generalize to other groups where different issues may impact decisions [14, 15]. Also, the average age in the sample is older than the typical Canadian perinatal woman (national average 30.8 years [42]) and we excluded women under age 18 so our results may therefore be less generalizable to young mothers. We did not have information on prior experiences with psychotherapy as this is not easily captured in quantitative variables; we did, however, have information on current engagement in psychotherapy and on past medication experiences, which is particularly germane to the decision of whether to use medication in pregnancy. We used logistic regression for our analyses, and results are therefore expressed as odds ratios. Since intent to take antidepressants is not a rare outcome, the odds ratio may not always closely approximate relative risk, and the point estimates should be interpreted accordingly. Finally, this study’s cross-sectional design means that we were only able to examine intent of whether to take medication. Longitudinal follow-up of preconception women to understand how decision trajectories evolve over time is likely warranted given the findings of the current study.

## Conclusion

This study highlights some of the unique considerations when considering how best to support women making antidepressant-related decisions while planning a pregnancy. Further research to design and evaluate additional ways to support preconception and pregnant women in their decision-making will help ensure that all such women have the opportunity to make well-informed decisions about their depression treatment, optimizing their own, and their child’s, health.

## Supplementary information

**Additional file 1: Table S1.** Current antidepressant medications used by participants (preconception women *n* = 55, pregnant women *n* = 40). **Figure S1.** Proportions of preconception (*n* = 55) and pregnant (*n* = 40) women who intend to use antidepressant medications in pregnancy, presented for the total cohort, and the cohort stratified by: age (< 35 years and ≥ 35 years), parity (nulliparous and primiparous/multiparous), and EPDS (< 13 and ≥ 13).

## Data Availability

The datasets used and/or analysed during the current study are available from the corresponding author on reasonable request.

## Abbreviations

DCS: Decisional Conflict Scale
EPDS: Edinburgh Postnatal Depression Scale
SNRI: Serotonin-norepinephrine reuptake inhibitor
SSRI: Selective serotonin reuptake inhibitor
STAI: State-Trait Anxiety Inventory
