# Supplementary material for: Decision-making about antidepressant medication use in pregnancy: a comparison between women making the decision in the preconception period versus in pregnancy
Source: BMC Psychiatry. 2020 Feb 7;20:54. doi: 10.1186/s12888-020-2478-8 (PMC7007680; doi:10.1186/s12888-020-2478-8)
Supplement: Supplementary file 1 — Additional file 1: Table S1. Current antidepressant medications used by participants (preconception women n = 55, pregnant women n = 40). Figure S1. Proportions of preconception (n = 55) and pregnant (n = 40) women who intend to use antidepressant medications in pregnancy, presented for the total cohort, and the cohort stratified by: age (< 35 years and ≥ 35 years), parity (nulliparous and primiparous/multiparous), and EPDS (< 13 and ≥ 13). [file 12888_2020_2478_MOESM1_ESM.docx]

**Table S1.** Current antidepressant medications used by participants (preconception women n=55, pregnant women n=40).

| **Current antidepressant medication** | **Preconception**  **(n=55)** | **Pregnant**  **(n=40)** |
| --- | --- | --- |
| ***Selective serotonin reuptake inhibitors (SSRIs)*** |  |  |
| Citalopram, n (%) | 5 (9.1) | 3 (7.5) |
| Escitalopram, n (%) | 18 (32.7) | 5 (12.5) |
| Fluoxetine, n (%) | 4 (7.3) | 1 (2.5) |
| Paroxetine, n (%) | 1 (1.2) | 0 (0) |
| Sertraline, n (%) | 11 (20.0) | 4 (10.0) |
| ***Serotonin and norepinephrine reuptake inhibitors (SNRIs)*** |  |  |
| Desvenlafaxine, n (%) | 3 (5.4) | 1 (2.5) |
| Venlafaxine, n (%) | 4 (7.3) | 2 (5.0) |
| ***Other*** |  |  |
| Buproprion, n (%) | 4 (7.3)^a^ | 2 (5.0) |
| Aripiprazole, n (%) | 3 (5.4) **^b^** | 0 (0) |
| ***No current antidepressant medication, n (%)*** | 8 (14.5) | 22 (55.0) |

^a^One preconception woman was taking buproprion as monotherapy, and three were taking buproprion in combination with an SSRI/SNRI. Two preconception women were taking buproprion as monotherapy.

^b^Three preconception women were taking aripiprazole in combination with an SSRI/SNRI, and none were taking aripiprazole as monotherapy. No pregnant women were taking aripiprazole.

**Figure S1.** Proportions of preconception (n=55) and pregnant (n=40) women who intend to use antidepressant medications in pregnancy, presented for the total cohort, and the cohort stratified by: age (<35 years and ≥35 years), parity (nulliparous and primiparous/multiparous), and EPDS (<13 and ≥13).

EPDS= Edinburgh Postnatal Depression Scale
